# Safety, Immunogenicity of Co-Administered Vaccines, and Lot-to-Lot Consistency of a 14-Valent Pneumococcal Conjugate Vaccine (PNEUBEVAX 14^®^) Administered at 6–10–14 Weeks in Healthy Infants: A Multicenter, Phase IV Trial

**DOI:** 10.3390/vaccines14060464

**Published:** 2026-05-22

**Authors:** Subhash Thuluva, Subbareddy Gunneri, Siddalingaiah Ningaiah, Vijay Yerroju, Rammohan Reddy Mogulla, Kamal Thammireddy, Chirag Dhar, Shivani Desai, Piyush Paliwal, Chandrudu Loka, Nagaganesh Balne, Suresh Kommanapalli, Chinmayi Joshi, Kishori Sharan Agarwal, Girish P. Charde, Manish Narang, Jai Prakash Narayan, Bheemisetty S. Chakravarthy, Niranjana S. Mahantshetti, Pramod Prabhakar Jog, Prashanth Madapura Virupakshappa, Savita Verma, Madhukar Pandey, Pareshkumar A. Thakkar

**Affiliations:** 1 Clinical Development, Biological E. Limited, 18/1&3, Azamabad, Hyderabad 500020, Indiasiddalingaiah.ningaiah@biologicale.com (S.N.);; 2 Belagavi Institute of Medical Sciences, Belgaum 590001, India; 3 Chirayu Hospital, Jaipur 302044, India; 4 Gillurkar Multispeciality Hospital, Nagpur 440024, India; 5 Guru Teg Bahadur Hospital, North East, Delhi 110095, India; 6 Jawahar Lal Nehru Medical College, Ajmer 305001, India; 7 King George Hospital, Visakhapatnam 530002, India; 8 Karnatak Lingayat Education Society Dr. Prabhakar Kore Hospital & Medical Research Centre, Belgaum 590010, India; 9 Medipoint Hospital, Pune 411007, India; 10 Medstar Speciality Hospital, Bangalore 560092, India; 11 Pandit Bhagwat Dayal Sharma Post Graduate Institute of Medical Sciences, Rohtak 124001, India; 12 Shubham Sadbhawana Superspeciality Hospital, Varanasi 221005, India; 13 Sir Sayajirao General Hospital, Vadodara 390001, India

**Keywords:** pneumococcal conjugate vaccine, PNEUBEVAX 14, BE-PCV14, 14-valent PCV, infant immunization, co-administration, routine vaccines, safety, immunogenicity, phase IV study

## Abstract

**Background:** Pneumococcal conjugate vaccines (PCVs) have substantially reduced pneumococcal disease in children; however, serotype distribution varies geographically, and residual disease due to non-PCV13 serotypes persists. Biological E’s PNEUBEVAX 14^®^ (BE-PCV14), a WHO-prequalified 14-valent PCV, expands coverage by including serotypes 22F and 33F. As PCVs are co-administered with routine Expanded Programme on Immunization (EPI) vaccines, post-licensure data on safety, co-administration, and lot-to-lot consistency are essential. This multicenter phase IV study evaluated BE-PCV14 in healthy PCV-naïve infants aged 6–8 weeks across 31 sites in India. **Methods:** A total of 2600 infants were enrolled and vaccinated at 6, 10, and 14 weeks of age; 2300 received BE-PCV14 and 300 received PCV13. All participants received concomitant DTwP-HepB-IPV-Hib and oral rotavirus vaccines per routine schedule. Safety was assessed through solicited and unsolicited adverse events (AEs) and serious adverse events (SAEs). Immunogenicity subsets evaluated responses to co-administered vaccines and serotype-specific responses across three BE-PCV14 lots. **Results:** Among 2600 vaccinated infants, at least one AE occurred in 26.35% (95% CI: 24.59, 28.19) of BE-PCV14 and 24.67% (95% CI: 20.13, 29.84) of PCV13 recipients; most were mild. Injection-site pain and pyrexia were the most common events. Immune responses to co-administered vaccines were comparable between groups and met the non-inferiority criteria: lower bound of the two-sided 95% CI > −10 percentage points for seroprotection/seroconversion rate differences using the Farrington–Manning method. Lot-to-lot consistency was demonstrated, with all GMC ratios within the predefined equivalence margin (0.5–2.0). **Conclusions:** BE-PCV14 was well tolerated. Immune responses to co-administered routine EPI vaccines met predefined non-inferiority criteria, supporting the interpretation that BE-PCV14 did not result in clinically meaningful immune interference. Consistent immune responses across manufacturing lots further support its use in infant immunization programs.

## 1. Introduction

*Streptococcus pneumoniae* remains a leading cause of pneumonia, meningitis, and sepsis in young children, with the highest burden of morbidity and mortality occurring in low- and middle-income countries. The introduction of pneumococcal conjugate vaccines (PCVs) into routine infant immunization programs has substantially reduced vaccine-type invasive pneumococcal disease (IPD) and pneumococcal mortality and is widely recommended by the World Health Organization (WHO) [1,2,3]. However, a substantial residual burden of pneumococcal disease remains, and serotype distribution continues to evolve in the post-PCV10 and post-PCV13 era [2,4]. Multi-country analyses and systematic reviews have highlighted the increasing contribution of non-PCV13 serotypes to residual IPD and the rationale for higher-valency PCVs [5,6]. In particular, serotypes 22F and 33F have emerged as important contributors to IPD beyond PCV13 in several regions [7,8] and have therefore been included in expanded-valency PCV formulations [9,10]. Indian data likewise list 22 F and 33 F among the ten most common non-vaccine serotypes causing pediatric IPD [11,12]. In routine practice, PCVs are administered during the same visit as other infant Expanded Programme on Immunization (EPI) vaccines [1,13]. Available evidence indicates that concomitant administration of PCVs with monovalent or combination vaccines containing diphtheria, tetanus, pertussis, hepatitis B, poliovirus, *Haemophilus influenzae* type b, and rotavirus antigens does not result in clinically meaningful effects on immunogenicity or reactogenicity. Nevertheless, WHO technical guidance emphasizes the importance of accumulating safety and immunogenicity data for co-administration with other infant vaccines and highlights the relevance of evaluating new PCVs on early infant schedules such as 6, 10, and 14 weeks, in which immune responses may be lower than with more widely spaced schedules [14]. Quantitative schedule-comparison studies support this concern: Spijkerman et al. reported higher post-primary PCV13 GMCs with a 2–4–6-month schedule than with a 2–3–4-month schedule for several serotypes [15], and Jones et al. reported that a later third PCV7 dose at 40 weeks produced GMCs generally 4–5-fold higher than those observed after a historical 6–10–14-week schedule [16].

Biological E’s 14-valent pneumococcal conjugate vaccine (PNEUBEVAX 14^®^; BE-PCV14) was developed to expand serotype coverage beyond PCV13 by including serotypes 22F and 33F. BE-PCV14 has been evaluated previously in randomized phase III studies in infants [10,17,18] and has subsequently received WHO prequalification [19]. However, post-licensure evidence remains important to expand the safety database and to evaluate the safety of concomitant administration and immune responses to co-administered vaccines in settings reflective of routine immunization programs. In addition, assessment of lot-to-lot consistency is important to show that commercial-scale vaccine lots elicit comparable immune responses [14].

Accordingly, this prospective, multicenter, phase IV study in healthy Indian infants vaccinated at 6, 10, and 14 weeks of age was conducted to evaluate: (i) the safety, tolerability, and reactogenicity of BE-PCV14 when administered concomitantly with routine EPI vaccines; (ii) immune responses to co-administered DTwP-HepB-IPV-Hib and oral rotavirus vaccines when given with BE-PCV14 compared with concomitant PCV13 administration; and (iii) post-primary serotype-specific pneumococcal IgG responses across three consecutively manufactured BE-PCV14 lots.

## 2. Materials and Methods

### 2.1. Study Design and Participants

This prospective, multicenter, open-label, phase IV study was conducted at 31 sites in India. Healthy infants aged 6–8 weeks at first vaccination were eligible if they were pneumococcal conjugate vaccine (PCV)-naïve and written informed consent was provided by a parent or legally acceptable representative. Key inclusion and exclusion criteria are provided in the Supplementary Material S1.

**Figure 1:**
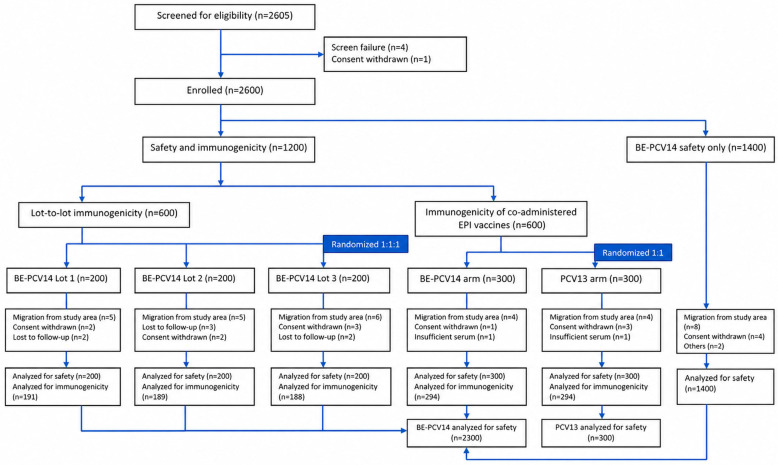
Participant disposition and study cohorts.

The study comprised two parallel cohorts (Figure 1), with cohort enrollment determined by prespecified site-based allocation. The safety-only cohort enrolled 1400 infants, all of whom received BE-PCV14. The immunogenicity-and-safety cohort enrolled 1200 infants and included two predefined subcohorts. In the first subcohort, 600 infants were randomized in a 1:1 ratio to receive either BE-PCV14 or PCV13 for evaluation of immune responses to co-administered vaccines, with 300 infants in each group. In the second subcohort, 600 infants were randomized in a 1:1:1 ratio to receive one of three consecutively manufactured BE-PCV14 lots for lot-to-lot consistency evaluation, with 200 infants per lot. Overall, 2300 infants received BE-PCV14 and 300 received PCV13. This unequal allocation was intentional because the primary purpose of the study was to expand the safety database for BE-PCV14, while PCV13 served only as an active comparator in the co-administered vaccine immunogenicity subcohort. The study was therefore not designed as a balanced comparative safety trial between BE-PCV14 and PCV13. Safety findings were evaluated descriptively, with the larger BE-PCV14-exposed population providing the main safety evidence.

All participants received a 3-dose primary series at approximately 6, 10, and 14 weeks of age. BE-PCV14 or PCV13 (0.5 mL) was administered intramuscularly into the anterolateral thigh. At the same visit, participants also received DTwP-HepB-IPV-Hib intramuscularly in the contralateral thigh and oral rotavirus vaccine according to the national immunization schedule. There was no patient or public involvement in designing, conducting, or reporting this study. Although the overall study included a booster phase, this report presents post-primary immunogenicity data through Day 84 and safety data through 6 months after the primary series.

BE-PCV14, Biological E’s 14-valent pneumococcal conjugate vaccine; PCV13, 13-valent pneumococcal conjugate vaccine; EPI, Expanded Programme on Immunization.

### 2.2. Ethics and Registration

The study protocol was approved by the institutional ethics committees at all 31 SAATHI-14 study sites, which are listed in the Supplementary Material S2. The protocol was also approved by the Central Drugs Standard Control Organisation (CDSCO), India’s National Regulatory Authority, and was prospectively registered with the Clinical Trials Registry-India (CTRI/2023/09/057894; registered on 20 September 2023). Written informed consent was obtained from a parent or legally acceptable representative of each participant before enrollment. The study was conducted in accordance with the Declaration of Helsinki, International Council for Harmonization Good Clinical Practice (ICH-GCP) guidelines, and applicable regulatory requirements.

### 2.3. Randomization and Masking

After informed consent was obtained, eligible participants were enrolled into one of two study cohorts at screening according to prespecified site-based allocation. The safety cohort was non-randomized, and all participants in this cohort received BE-PCV14. In the immunogenicity-and-safety cohort, participants in the co-administered vaccine immunogenicity subset were randomized 1:1 to receive BE-PCV14 or PCV13, whereas those in the lot-to-lot consistency subset were randomized 1:1:1 to receive one of three consecutively manufactured BE-PCV14 lots. Randomization was implemented using a validated interactive web response system (IWRS). The study was open-label; therefore, investigators and participants’ parents or legally acceptable representatives were aware of the assigned vaccine. The open-label design was selected because of the pragmatic Phase IV design, differing cohort objectives, unequal allocation, site-based cohort assignment, and vaccine allocation across comparator and lot groups, which made full blinding operationally infeasible. Potential bias was minimized through a common visit schedule, standardized diary cards, predefined adverse-event grading criteria, and uniform safety reporting procedures. Laboratory personnel performing immunogenicity assays were blinded to treatment group assignment.

The randomized components of the study were reported in accordance with the CONSORT guidelines, as applicable.

### 2.4. Procedures

Screening assessments included medical history, physical examination, and general health evaluation. Study visits were scheduled at enrollment/first vaccination (Day 0), dose 2 (Day 28), dose 3 (Day 56), post-primary follow-up (Day 84), and a 6-month safety follow-up after dose 3.

Participants received BE-PCV14 or PCV13 intramuscularly in the anterolateral thigh. At the same visit, all participants also received DTwP-HepB-IPV-Hib intramuscularly in the contralateral thigh and oral rotavirus vaccine according to the national immunization schedule. Detailed vaccine compositions are provided in the Supplementary Material S3. The three BE-PCV14 lots used for lot-to-lot consistency assessment were consecutively manufactured finished-product lots produced using the same validated manufacturing process, final formulation, antigen content, CRM_197_ conjugation platform, adjuvant, preservative, and predefined release specifications. The final composition was identical across lots, and all lots met predefined quality-control and batch-release specifications before clinical use.

Participants were observed for at least 30 min after each vaccination for immediate adverse reactions. Parents or legally acceptable representatives were provided with diary cards to record solicited local and systemic adverse events (AEs) occurring within 7 days after vaccination. Solicited adverse events were collected using standardized, structured diary cards with predefined event terms, reporting time windows, and grading instructions. Parents or legally acceptable representatives were trained by site staff at each vaccination visit on how to record solicited symptoms, including use of a thermometer for temperature measurement and, where applicable, measurement of injection-site erythema and swelling. Diary cards were reviewed by study staff at subsequent visits to ensure completeness and consistency of interpretation, and unclear entries were clarified with the parent or legally acceptable representative using prespecified definitions.

Unsolicited AEs were recorded through Day 84, and serious adverse events (SAEs) were recorded throughout the study period. AE intensity was graded using the Common Terminology Criteria for Adverse Events (CTCAE), version 5.0, and the Division of AIDS (DAIDS) Table for Grading the Severity of Adult and Pediatric Adverse Events, version 2.0, as applicable. Febrile events were graded according to Brighton Collaboration criteria. Causality was assessed by the study investigator.

For immunogenicity assessments, peripheral venous blood was collected before dose 1 and 28 days after dose 3 from the relevant participant subsets. Samples were processed and stored according to predefined laboratory procedures until analysis.

Serotype-specific anti-pneumococcal capsular polysaccharide (PnCPS) IgG concentrations against all 14 vaccine serotypes were measured using the WHO reference enzyme-linked immunosorbent assay (ELISA), with a minor modification. The details are provided in the Supplementary Material S4. Cell wall polysaccharide (CWPS) multi was used instead of CWPS plus 22F pneumococcal polysaccharide for pre-adsorption to neutralize non-specific antibodies [20]. Immune responses to co-administered vaccines were assessed using validated assays for diphtheria, tetanus, hepatitis B, *Haemophilus influenzae* type b, pertussis antigens, poliovirus types 1, 2, and 3, and rotavirus. Additional assay details, kits and testing laboratories are provided in the Supplementary Material S4. Although the study included a PCV13 group within the co-administration cohort, the direct comparison of pneumococcal immune responses between BE-PCV14 and PCV13 was not an objective of the present analysis. Those data are part of a separate 3 + 1 immunogenicity evaluation and are reported separately [21].

### 2.5. Outcomes

The primary objective was to evaluate the safety, tolerability, and reactogenicity of BE-PCV14 administered as a 3-dose primary series together with routine EPI vaccines. The primary safety analysis focused on events reported through Day 84 (28 days after dose 3).

Secondary objectives were to assess immune responses to co-administered routine vaccines, describe the consistency of serotype-specific immune responses across three BE-PCV14 manufacturing lots, and summarize safety through the 6-month follow-up after the primary series.

### 2.6. Sample Size Determination

The primary objective of this phase IV study was to expand the safety database for BE-PCV14 and further characterize its safety profile in infants. In line with WHO guidance recommending a safety database of approximately 3000 exposed participants, and because 995 infants had received BE-PCV14 in earlier phase III studies [10,17,18], an additional 2300 infants were enrolled in the present study, resulting in a cumulative BE-PCV14 safety database of more than 3000 exposed participants.

The co-administered vaccine immunogenicity subcohort was predefined to include 600 infants randomized 1:1 to BE-PCV14 or PCV13, with 300 participants per group. Assuming no immune interference, comparable response rates between groups, a one-sided alpha of 2.5%, and a −10 percentage-point non-inferiority margin for seroprotection/seroconversion rate differences, 300 participants per group provided approximately 98% power for each individual comparison when the expected response rate was 90%.

The lot-to-lot consistency subcohort was predefined to include 600 infants randomized 1:1:1 to receive one of three consecutively manufactured BE-PCV14 lots, with 200 participants per lot. Assuming a true GMC ratio of 1.0 between lots, a common standard deviation of 2.0 on the log-transformed antibody scale, a one-sided alpha of 2.5%, and an equivalence margin of 0.5 to 2.0 for the GMC ratio, approximately 175 evaluable participants per lot were required to provide 90% power for each individual pairwise serotype comparison. Allowing for a 10% non-evaluable rate, approximately 195 participants per lot were required; therefore, the planned sample size of 200 participants per lot was considered adequate.

### 2.7. Statistical Analysis

Demographic and baseline characteristics were summarized descriptively for the safety population, defined as all participants who received at least one dose of the study vaccine. Safety analyses were performed in this population. Immunogenicity analyses were performed in the per-protocol (PP) population, defined as participants who received all three doses within protocol-defined windows, had evaluable immunogenicity results, and had no major protocol deviations affecting immunogenicity and or safety assessment.

Continuous variables were summarized using the number of observations, mean, standard deviation, and median, and categorical variables using frequencies and percentages. Adverse events were summarized descriptively by system organ class (SOC) and preferred term (PT). Two-sided 95% confidence intervals (CIs) for proportions were calculated using the Wilson score method. Adverse events were coded using MedDRA version 25.1.

For the co-administered vaccine immunogenicity evaluation, seroprotection rates were calculated for diphtheria, tetanus, hepatitis B, *Haemophilus influenzae* type b, and poliovirus types 1, 2, and 3, and seroconversion rates were calculated for pertussis antigens and rotavirus. Geometric mean concentrations/titers (GMCs/GMTs) were estimated for all co-administered vaccine antigens. In addition to the prespecified descriptive analyses, a non-inferiority analysis was also performed. The between-group difference in seroprotection or seroconversion rates and its two-sided 95% CI were calculated using the Farrington–Manning method. Non-inferiority of BE-PCV14 relative to PCV13 was concluded if the lower bound of the two-sided 95% CI for the between-group difference was greater than −10 percentage points. For continuous endpoints, analyses were based on a two-sample Student’s *t*-test on log-transformed values, with back-transformation for presentation as GMC/GMT ratios and corresponding two-sided 95% CIs. For GMC/GMT ratios (BE-PCV14/PCV13), non-inferiority was concluded if the lower bound of the two-sided 95% CI was greater than 0.5. The non-inferiority margins were selected based on criteria used in prior PCV15 and PCV20 infant trials evaluating immune responses to co-administered vaccine antigens [9,22]. The −10 percentage-point margin for seroprotection/seroconversion rate differences was selected to exclude a clinically meaningful reduction in the proportion of infants achieving accepted immune-response thresholds for the co-administered antigens. The GMC/GMT ratio criterion requiring the lower bound of the two-sided 95% CI to be greater than 0.5 was selected to exclude a substantial reduction in antibody concentrations or titers to the co-administered antigens.

For the lot-to-lot consistency evaluation, the proportions of participants with serotype-specific IgG concentrations ≥0.35 µg/mL and serotype-specific IgG GMCs at Day 84 were calculated for each vaccine serotype in each lot group, together with two-sided 95% CIs. Antibody concentrations were log-transformed for GMC analyses and back-transformed for presentation. Pairwise GMC ratios with two-sided 95% CIs were calculated across the three BE-PCV14 lots. The lot-to-lot equivalence was concluded if each pairwise GMC ratio and its two-sided 95% CI lay entirely within 0.5 to 2.0 [23,24].

No adjustment for multiplicity was applied. Therefore, nominal *p*-values and isolated between-group differences were interpreted descriptively and should not be considered confirmatory evidence of superiority or enhanced immunogenicity. Missing data were not imputed, and all analyses were performed using observed data only. Statistical analyses were conducted using SAS^®^ version 9.4 (SAS Institute Inc., Cary, NC, USA).

### 2.8. Data Management and Independent Statistical Analysis

Clinical data management and database validation were performed by an external data management vendor in accordance with a predefined data management plan. The database was locked prior to analysis. Statistical analyses were performed by an independent statistician (not employed by the sponsor) using the locked dataset and the predefined statistical analysis plan. The sponsor and study team reviewed the results for clinical interpretation; however, data handling, database lock and statistical analyses were performed independently to support objectivity. The data was reviewed by the independent Data Safety Monitoring Board.

## 3. Results

The study was conducted at 31 sites across India between 14 February 2024 and 16 June 2025.

A total of 2605 healthy infants were screened; four failed screening, and one withdrew consent before vaccination. Overall, 2600 participants were vaccinated and included in the safety population; 2300 received BE-PCV14 and 300 received PCV13 (Figure 1). Safety analyses were performed in the safety population. The per-protocol population comprised 588 participants (98.0%) in the co-administered vaccine immunogenicity subset, including 294 participants in each study group, and 568 participants (94.7%) in the lot-to-lot consistency subset with evaluable immunogenicity data (Figure 1). Baseline demographic and anthropometric characteristics are summarized in Table 1 and were broadly similar between groups. The mean age at enrollment was approximately 48 days, mean length was approximately 54 cm, and median body weight was approximately 4.0 kg in both groups. Female infants comprised 44.0% of the BE-PCV14 group and 42.0% of the PCV13 group. All participants received DTwP-HepB-IPV-Hib and rotavirus vaccine concomitantly with PCVs according to the routine immunization schedule.

### 3.1. Safety, Tolerability, and Reactogenicity Findings

**Figure 2:**
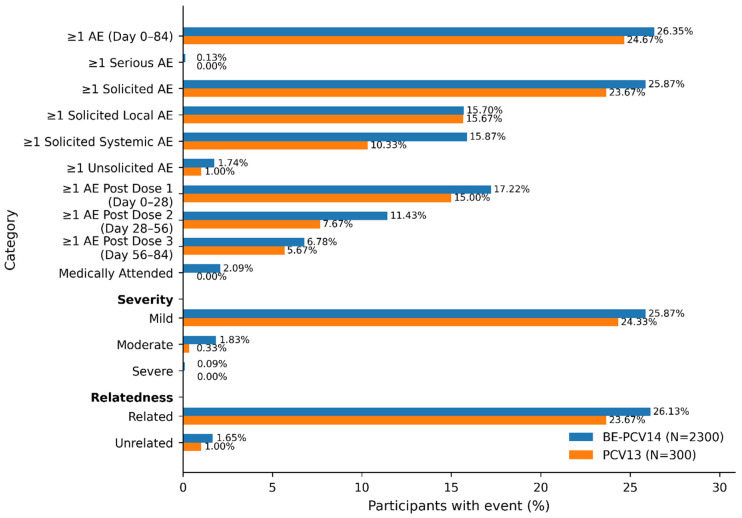
Overview of participants reporting adverse events from day 0 through day 84 (N = 2600). AE: Adverse event, %: percentage.

The primary objective of the study was a descriptive evaluation of safety through Day 84, corresponding to 28 days after completion of the 3-dose primary series. Safety outcomes were summarized descriptively by treatment group, and no formal statistical testing was performed for between-group safety comparisons. An overview of adverse events reported through Day 84 is presented in Figure 2, and detailed summaries are provided in the Supplementary Material S5. Through Day 84, at least one adverse event was reported in 606 of 2300 participants (26.35%; 95% CI: 24.59, 28.19) in the BE-PCV14 group and in 74 of 300 participants (24.67%; 95% CI: 20.13, 29.84) in the PCV13 group. Most events were solicited, reported during the 7-day post-vaccination diary period, and mild in intensity. The proportions of participants reporting adverse events after doses 1, 2, and 3 were 17.22%, 11.43%, and 6.78%, respectively, in the BE-PCV14 group and 15.00%, 7.67%, and 5.67%, respectively, in the PCV13 group. Severe adverse events were uncommon and were reported in two participants (0.09%) in the BE-PCV14 group. Through Day 84, three participants (0.13% [95% CI: 0.04, 0.38]) reported five serious adverse events.

Solicited adverse events occurring within 7 days after vaccination are summarized in Table 2. Across Day 0 to Day 84, 595 participants (25.87%; 95% CI: 24.12, 27.70) reported 1190 solicited adverse events in the BE-PCV14 group, and 71 participants (23.67%; 95% CI: 19.21, 28.79) reported 90 solicited adverse events in the PCV13 group. In the BE-PCV14 group, the most frequently reported local solicited adverse events were injection-site pain (11.00%), swelling (6.35%), erythema (4.52%), and induration (1.78%), whereas the most frequently reported systemic solicited adverse events were pyrexia (13.83%) and post-vaccination irritability (2.96%). Ten participants (0.43%; 95% CI: 0.24, 0.80) reported 11 adverse events within 30 min after vaccination, including pyrexia, injection-site pain, injection-site erythema, and post-vaccination irritability. No hypersensitivity or anaphylactic reactions were reported.

Unsolicited adverse events through Day 84 were uncommon and are summarized in the Supplementary Material S6. In the BE-PCV14 group, 63 unsolicited adverse events were reported in 40 participants (1.74%; 95% CI: 1.28, 2.36), with infections and infestations being the most frequently reported system organ class (29 events in 24 participants [1.04%]).

During the 6-month follow-up after the primary series, 74 events were reported in 59 of 2300 participants (2.57%; 95% CI: 1.99, 3.29) in the BE-PCV14 group and five events were reported in five of 300 participants (1.67%; 95% CI: 0.71, 3.84) in the PCV13 group (Supplementary Material S7). In the BE-PCV14 group, the most frequently reported adverse events during follow-up were pyrexia, vomiting, nasopharyngitis, and diarrhoea. In the PCV13 group, pyrexia and cough were the most frequently reported events. Most events were mild, and no new safety signal was identified. Moderate adverse events were reported in 1.83% of participants in the BE-PCV14 group and 0.33% in the PCV13 group, whereas severe adverse events were reported only in the BE-PCV14 group (0.09%).

A summary of serious adverse events is presented in the Supplementary Materials S8 and S9. Six serious adverse events were reported in four of 2300 participants, all in the BE-PCV14 group, over the entire study period, including the 6-month follow-up after primary series (0.17%; 95% CI: 0.05, 0.44). Five serious adverse events were reported by three participants (0.13%; 95% CI: 0.04, 0.38) between Day 0 and Day 84, including two events of acute gastroenteritis, two events of dehydration, and one event of viral pneumonia. One serious adverse event of lower respiratory tract infection with pneumonitis occurred during the 6-month follow-up. There were no severe adverse events reported among the 300 PCV13 recipients. All serious adverse events were assessed by the investigators as unrelated to study vaccination, and the Data Safety Monitoring Board (DSMB) concurred with these causality assessments during its safety review.

### 3.2. Immunogenicity of Co-Administered EPI Vaccines

Immune responses to routine EPI vaccine antigens were evaluated after concomitant administration with either BE-PCV14 or PCV13 to assess potential immune interference. Immune responses to diphtheria, tetanus, hepatitis B, *Haemophilus influenzae* type b, poliovirus types 1, 2, and 3, pertussis antigens, and rotavirus were assessed at Day 84, corresponding to 28 days after completion of the 3-dose primary series. Among the 600 infants enrolled in this subset, 588 (98.0%) comprised the per-protocol population, with evaluable Day 84 immunogenicity data available for 294 participants in each study group.

Seroprotection/seroconversion rates to co-administered vaccine antigens were high and similar between groups (Table 3). Seroprotection rates for diphtheria, tetanus, hepatitis B, and *H. influenzae* type b were 98.6%, 99.7%, 91.5%, and 98.0%, respectively, in the BE-PCV14 group and 96.6%, 99.3%, 93.9%, and 98.6%, respectively, in the PCV13 group. Seroprotection or seroconversion rates for poliovirus types 1, 2, and 3 ranged from 90.8% to 94.9% across the two groups. Pertussis seroconversion rates were 84.7% and 86.1% for agglutination and 66.7% and 64.6% for pertussis toxin in the BE-PCV14 and PCV13 groups, respectively. Rotavirus IgA responses were 98.6% in the BE-PCV14 group and 98.9% in the PCV13 group. The non-inferiority criteria for seroprotection or seroconversion rates were met for all evaluated antigens (Table 3 and Supplementary Material S10). GMCs/GMTs for co-administered vaccine antigens were also comparable between groups (Table 3). The non-inferiority criteria based on GMC/GMT ratios were met for all evaluated antigens, with the lower bound of each two-sided 95% confidence interval greater than 0.5 (Table 3, Supplementary Material S10). These findings suggest that concomitant administration of DTwP-HepB-IPV-Hib and rotavirus vaccines with BE-PCV14 did not result in clinically meaningful immune interference.

### 3.3. Lot-to-Lot Immunogenicity Consistency of BE-PCV14

Serotype-specific IgG responses were evaluated in a subset of infants randomized to receive one of three consecutively manufactured commercial-scale lots of BE-PCV14. Of the 600 infants enrolled in this subset, 568 (94.7%) comprised the per-protocol population, with valid paired immunogenicity data available for 191 of 200 participants (95.5%) in Lot 1, 189 of 200 (94.5%) in Lot 2, and 188 of 200 (94.0%) in Lot 3.

Day 84 serotype-specific IgG GMCs were generally similar across the three BE-PCV14 lots (Table 4). All pairwise GMC-ratio 95% confidence intervals for the 14 vaccine serotypes lay entirely within the predefined equivalence margin of 0.5 to 2.0 (Table 4 and Supplementary Material S11). Pairwise GMC ratios ranged from 0.94 to 1.75 for Lot 1 versus Lot 3, 0.94 to 1.38 for Lot 1 versus Lot 2, and 1.05 to 1.36 for Lot 2 versus Lot 3, with all corresponding confidence intervals remaining within the equivalence bounds. Together, these results were consistent with comparable post-primary immune responses across the three consecutively manufactured BE-PCV14 lots. To improve visualization of between-lot variability, serotype-specific IgG GMC ratios were additionally normalized to Lot 1 as the reference lot. In this analysis, Lot 2/Lot 1 ratios ranged from 0.72 to 1.06, and Lot 3/Lot 1 ratios ranged from 0.57 to 0.97 across the 14 serotypes. Although Lot 3 showed numerically lower GMCs for several serotypes, all normalized ratios remained within the predefined equivalence margin of 0.5 to 2.0, supporting the conclusion of lot-to-lot consistency. (Supplementary Material S12).

## 4. Discussion

This multicenter phase IV study was conducted to expand the safety database for BE-PCV14 and to evaluate immune responses to concomitantly administered routine EPI vaccines, as well as lot-to-lot consistency of BE-PCV14. Although the safety and immunogenicity of BE-PCV14 relative to PCV13 had previously been shown in three phase III studies in infants [10,17,18], the present study adds substantial safety experience from 2300 participants and extends follow-up to 6 months after completion of the 3-dose primary series.

Overall, BE-PCV14 was well tolerated, with no new safety concerns identified. Most adverse events were mild to moderate, largely solicited, and occurred within 7 days after vaccination. Immediate adverse events within 30 min after vaccination were infrequent, occurring in 0.43% of BE-PCV14 recipients, and no hypersensitivity or anaphylactic reactions were reported. Although most published PCV trials have not specifically described adverse events within 30 min after vaccination, available regulatory assessment documents suggest that such events may occur at low frequency in clinical development programs, with reported incidences ranging from ≤0.7% to 4.6% [25,26].

The observed safety profile was characterized mainly by expected local reactions, particularly injection-site pain, swelling, erythema, and induration, and by systemic events such as pyrexia and post-vaccination irritability.

Unsolicited adverse events through Day 84 were uncommon. Infections and infestations were the most frequently reported system organ class. This pattern may be consistent with intercurrent illnesses commonly observed during infancy, as reported in Indian birth-cohort and community-based studies; however, no definitive attribution to background rates can be made because the present study was not designed to formally compare observed event rates with background incidence rates [27,28].

During the 6-month follow-up after the third dose, adverse events remained infrequent and were largely limited to pyrexia, respiratory tract infections, and gastrointestinal illnesses commonly encountered in this age group. No clustering of late-onset adverse events or unexpected findings was observed. These findings are consistent with earlier phase III studies of BE-PCV14 [10,17,18] and with the established safety profile of other pneumococcal conjugate vaccines in infants [17,22,24,29], in which transient local reactogenicity and short-lived systemic events such as pyrexia and irritability are commonly observed. In the comparison trial of PCV20 vs. PCV13 in infants ≥ 1 AE was reported in 36.6% of participants in the PCV20 group and 39.4% of participants in the PCV13 group [22].

Severe adverse events were reported only among BE-PCV14 recipients; however, this finding should be interpreted in the context of the unequal group sizes, with 2300 infants receiving BE-PCV14 compared with 300 receiving PCV13. The study was not designed as a balanced comparative safety trial between BE-PCV14 and PCV13. The severe events were rare, were not considered related to study vaccination by the investigators, and did not show a consistent clinical pattern suggestive of a vaccine-related safety concern.

Because routine infant immunization schedules commonly involve the administration of multiple vaccines at the same visit, it is important to show that the addition of a pneumococcal conjugate vaccine does not adversely affect immune responses to co-administered antigens. In this study, immune responses to the co-administered hexavalent vaccine (DTwP-HepB-IPV-Hib) and oral rotavirus vaccine were similar between the BE-PCV14 and PCV13 groups, with robust responses observed for all evaluated antigens. Non-inferiority was established for seroprotection or seroconversion rates and GMC/GMT ratios. Some GMCs/GMTs for co-administered antigens were numerically higher in the BE-PCV14 group than in the PCV13 group. These differences should be interpreted cautiously because the co-administered injectable vaccine was given in the contralateral thigh, the rotavirus vaccine was administered orally, and there is no clear biological basis to infer immune enhancement by BE-PCV14. Moreover, multiple antigen-specific endpoints were evaluated without multiplicity adjustment; therefore, isolated nominal differences may reflect random variation. These findings suggest that concomitant administration of BE-PCV14 did not result in clinically meaningful immune interference and are consistent with previous studies of other PCVs in which no clinically meaningful interference with co-administered vaccines has been observed [9,22,24].

Lot-to-lot consistency is an important component of vaccine development because it helps show that commercial-scale manufacturing yields comparable immune responses across vaccine lots. In the present study, Day 84 serotype-specific GMCs were similar across the three BE-PCV14 lots. The 95% confidence intervals for all pairwise GMC ratios were fully contained within the equivalence margin of 0.5 to 2.0 for all vaccine serotypes, supporting comparable post-primary immune responses across the three evaluated lots. These findings are also in line with previous lot-to-lot consistency assessments reported during the clinical development of other pneumococcal conjugate vaccines [23,24]. GMCs for Lot 3 were numerically lower than those for Lots 1 and 2 for several serotypes; however, all pairwise GMC-ratio 95% CIs remained within the predefined equivalence margin of 0.5 to 2.0. No serotype-specific outlier or failure of equivalence criteria was observed. All three lots had identical final composition, were manufactured using the same validated process and release specifications, and met predefined quality-control and batch-release criteria before clinical use. The normalized Lot 1-referenced analysis also showed numerically lower GMCs for Lot 3 for several serotypes; however, these differences remained within the predefined equivalence margin and did not alter the conclusion of lot-to-lot consistency.

### Limitations

This study was primarily intended to expand the safety database of BE-PCV14 and to evaluate co-administration and lot-to-lot consistency. Several design limitations should be considered when interpreting the findings. The trial was open-label, which may have introduced reporting or ascertainment bias, particularly for subjective solicited adverse events. In addition, the safety population included unequal group sizes, with substantially more participants receiving BE-PCV14 than PCV13, and the BE-PCV14 safety database included a non-randomized safety cohort. These design features were driven by the primary objective of expanding the BE-PCV14 safety database and were not intended to support formal comparative safety conclusions between BE-PCV14 and PCV13. Therefore, safety findings should be interpreted descriptively, with emphasis on the overall safety profile of BE-PCV14 and the absence of new safety signals rather than on direct between-group comparisons.

The present manuscript does not include a direct pneumococcal immunogenicity comparison between BE-PCV14 and PCV13. Those data are part of a separate 3 + 1 immunogenicity evaluation and are reported separately [21]. Therefore, the present analysis should be interpreted in the context of its predefined objectives: safety database expansion, lot-to-lot consistency of BE-PCV14, and immune responses to co-administered routine vaccines. Immunogenicity assessment in the present study was limited to IgG responses and did not include functional antibody assessment. However, data from a pivotal phase III trial using a 3 + 0 schedule demonstrated comparable OPA responses between BE-PCV14 and PCV13 [10].

In addition, multiple immunogenicity endpoints were evaluated across several co-administered vaccine antigens and pneumococcal serotypes without adjustment for multiplicity; therefore, isolated nominal differences should be interpreted cautiously.

Finally, because the study enrolled only healthy PCV-naïve Indian infants aged 6–8 weeks, the findings may not be directly generalizable to other geographic or epidemiologic settings, preterm infants, infants with underlying medical conditions, or immunocompromised individuals. Additional studies or post-marketing data are needed to further characterize safety and immunogenicity in these populations.

## 5. Conclusions

BE-PCV14, administered as a 3-dose primary series at 6, 10, and 14 weeks of age, was well tolerated in healthy Indian infants, with no new safety signals identified through 6 months after completion of the primary series. The findings also support concomitant administration of BE-PCV14 with routine EPI vaccines, including DTwP-HepB-IPV-Hib and rotavirus vaccine, without evidence of clinically meaningful immune interference. Comparable immune responses were also observed across the three BE-PCV14 lots.

## Figures and Tables

**Table 1:** Demographic and baseline characteristics (N = 2600).

Characteristic	BE-PCV14	PCV13
Total number of participants in study	N = 2300	N = 300
Mean Age (±SD) in days	47.6 (±3.95)	48.2 (±3.80)
Mean Length (±SD) in centimeters	54.11 (±2.09)	53.68 (±1.46)
Median Weight (Range) in kilograms	4.05 (3.30–6.04)	4.10 (3.30–6.10)
Total number of male participants *n* (%)	1287 (55.96%)	174 (58.00%)
Total number of female participants *n* (%)	1013 (44.04%)	126 (42.00%)

N: Total number of participants, PCV: Pneumococcal conjugate vaccine, BE-PCV14: Biological E’s 14-valent PCV, PCV13: Pfizer’s 13-valent PCV, SD: Standard deviation.

**Table 2:** Summary of solicited local and systemic adverse events within 7 days post vaccination period by SOC & PT from dose 1 to 28 days post 3 dose primary series—safety population (N = 2600).

System Organ Class Preferred Term n (%) [95% CI] E	BE-PCV14 (N = 2300)		PCV13 (N = 300)	
	No. of participants with AE n (%) [95% CI]	Number of events [E]	No. of participants with AE n (%) [95% CI]	Number of events [E]
No. of participants with at least one solicited AE during Day 0 to Day 84	595 (25.87) [24.12, 27.70]	1190	71 (23.67) [19.21, 28.79]	90
Gastrointestinal disorders	19 (0.83) [0.53, 1.29]	20	0 [ NE ]	0
Diarrhoea	14 (0.61) [0.36, 1.02]	14	0 [ NE ]	0
Vomiting	6 (0.26) [0.12, 0.57]	6	0 [ NE ]	0
General disorders and administration site conditions	590 (25.65) [23.91, 27.48]	1141	71 (23.67) [19.21, 28.79]	90
Crying	1 (0.04) [0.01, 0.25]	1	0 [NE]	0
Injection site erythema	104 (4.52) [3.75, 5.45]	110	15 (5.00) [3.05, 8.08]	16
Injection site induration	41 (1.78) [1.32, 2.41]	46	0 [NE]	0
Injection site pain	253 (11.00) [9.79, 12.34]	313	26 (8.67) [5.98, 12.40]	26
Injection site swelling	146 (6.35) [5.42, 7.42]	174	12 (4.00) [2.30, 6.86]	15
Irritability postvaccinal	68 (2.96) [2.34, 3.73]	100	1 (0.33) [0.06, 1.86]	1
Pyrexia	318 (13.83) [12.48, 15.30]	397	31 (10.33) [7.38, 14.29]	32
Metabolism and nutrition disorders	22 (0.96) [0.63, 1.44]	25	0 [ NE ]	0
Decreased appetite	22 (0.96) [0.63, 1.44]	25	0 [ NE ]	0
Skin and subcutaneous tissue disorders	3 (0.13) [0.04, 0.38]	4	0 [ NE ]	0
Urticaria	3 (0.13) [0.04, 0.38]	4	0 [ NE ]	0

n: number of participants, %: percentage, CI: Confidence Interval, E: Event, NE: Not estimable. 95% CI was calculated using the Wilson score method.

**Table 3:** Percentage of participants seroprotected/seroconverted and geometric mean titres/concentrations for diphtheria, tetanus, hepatitis B, *haemophilus influenzae* type b, IPV, pertussis agglutinin, pertussis toxin, and rotavirus IgA concentration—PP population (N = 588).

Antigens	SPR/SCR % (95% CI)			GMTs/GMCs (95% CI)		
	BE-PCV14 (*n* = 294) *	PCV13 (*n* = 294) #	Difference in SPR/SCR BE-PCV14—PCV13 (95% CI)	BE-PCV14 (*n* = 294) *	PCV13 (*n* = 294) #	GMC Ratio BE-PCV14/PCV13 (95% CI)
Diphtheria (IU/mL)	98.6 (96.6, 99.5)	96.6 (93.9, 98.1)	+2.0 (−0.6, 4.9)	1.08 (0.96, 1.21)	0.83 (0.73, 0.95)	1.30 (1.08, 1.55)
Tetanus (IU/mL)	99.7 (98.1, 99.9)	99.3 (97.6, 99.8)	+0.3 (−1.3, 2.1)	1.32 (1.21, 1.45)	1.17 (1.06, 1.30)	1.13 (0.98, 1.29)
Hepatitis B (mIU/mL)	91.5 (87.7, 94.2)	93.9 (90.5, 96.1)	−2.4 (−6.7, 1.9)	834.34 (632.1, 1101.2)	629.4 (489.5, 809.4)	1.33 (0.91, 1.93)
Hib (µg/mL)	98.0 (95.6, 99.1)	98.6 (96.6, 99.5)	−0.7 (−3.2, 1.7)	4.55 (3.83, 5.41)	4.29 (3.65,5.04)	1.06 (0.84, 1.34)
Polio 1 (ED50 units)	93.5 (90.1, 95.8)	94.9 (91.8, 96.9)	−1.4 (−5.3, 2.5)	507.29 (423.3, 607.9)	482.76 (406.06, 573.96)	1.05 (0.82, 1.35)
Polio 2 (ED50 units)	93.9 (90.5, 96.1)	93.5 (90.1, 95.8)	+0.3 (−3.7, 4.4)	225.04 (191.2, 264.9)	167.27 (143.74, 194.65)	1.35 (1.08, 1.68)
Polio 3 (ED50 units)	90.8 (87.0, 93.6)	94.9 (91.8, 96.9)	−4.1 (−8.4, 0.1)	524.41 (428.3, 642.00)	459.66 (385.75, 547.72)	1.14 (0.87, 1.49)
Rota Ig A (U/mL)	98.6 (96.5, 99.5)	98.9 (97.0, 99.6)	−0.4 (−2.6, 1.8)	86.67 (80.4,93.4)	86.07 (79.86, 92.75)	1.01 (0.91, 1.12)
Pertussis aggl.	84.7 (80.1, 88.4)	86.1 (81.6, 89.6)	−1.4 (−7.1, 4.4)	141.32 (128.6, 155.3)	118.70 (106.48, 132.32)	1.19 (1.03, 1.38)
Pertussis PT (µg/mL)	66.7 (61.1, 71.8)	64.6 (59.0, 69.9)	+2.1 (−3.5, 7.7)	29.75 (26.1, 33.9)	28.02 (24.49, 32.06)	1.06 (0.88, 1.28)

For Rota IgA * n = 288 in BE-PCV14 arm and # n = 290 in PCV13 arm, aggl = agglutinin, PT = Pertussis Toxin, % = percentage, SPR: Seroprotection rate, SCR: Seroconversion rate, CI: Confidence Interval, BE-PCV14: 14-valent Pneumococcal conjugate vaccine from Biological E. Limited, PCV13: 13-valent Pneumococcal conjugate vaccine.

**Table 4:** Geometric mean concentrations at day 84 for serotype-specific anti-pneumococcal capsular polysaccharide antibody concentration (µg/mL)—per protocol population (N = 568).

Serotype	Lot 1 (N = 191) GMC * (95% CI)	Lot 2 (N = 189) GMC * (95% CI)	Lot 3 (N = 188) GMC * (95% CI)	Lot1/Lot2 GMC * Ratio (95% CI)	Lot1/Lot3 GMC * Ratio (95% CI)	Lot2/Lot3 GMC * Ratio (95% CI)
**1**	1.72 (1.50, 1.97)	1.76 (1.52, 2.03)	1.48 (1.30, 1.68)	0.98 (0.80, 1.19)	1.16 (0.97, 1.40)	1.19 (0.98, 1.44)
**3**	0.74 (0.66, 0.84)	0.71 (0.63, 0.81)	0.61 (0.54, 0.70)	1.04 (0.88, 1.24)	1.21 (1.01, 1.44)	1.16 (0.97, 1.39)
**4**	1.55 (1.36, 1.76)	1.62 (1.40, 1.86)	1.48 (1.29, 1.69)	0.96 (0.79, 1.16)	1.05 (0.87, 1.26)	1.09 (0.90, 1.33)
**5**	1.70 (1.48, 1.96)	1.82 (1.53, 2.16)	1.63 (1.42, 1.88)	0.94 (0.75, 1.17)	1.04 (0.86, 1.27)	1.11 (0.89, 1.39)
**6B**	1.56 (1.23, 1.97)	1.13 (0.90, 1.41)	0.89 (0.73, 1.08)	1.38 (1.00, 1.91)	1.75 (1.29, 1.98)	1.27 (0.94, 1.71)
**7F**	2.10 (1.83, 2.41)	2.19 (1.84, 2.61)	2.03 (1.74, 2.37)	0.96 (0.77, 1.20)	1.03 (0.84, 1.27)	1.08 (0.85, 1.36)
**9V**	2.20 (1.84, 2.64)	2.19 (1.84, 2.60)	1.71 (1.45, 2.03)	1.01 (0.78, 1.29)	1.29 (1.00, 1.65)	1.28 (1.00, 1.62)
**14**	8.60 (7.31, 10.12)	9.02 (7.88, 10.32)	7.73 (6.66, 8.96)	0.95 (0.77, 1.18)	1.11 (0.89, 1.39)	1.17 (0.96, 1.43)
**18C**	1.60 (1.37, 1.88)	1.71 (1.45, 2.01)	1.26 (1.05, 1.50)	0.94 (0.75, 1.18)	1.28 (1.01, 1.62)	1.36 (1.07, 1.73)
**19A**	3.58 (3.08, 4.16)	3.69 (3.19, 4.27)	2.96 (2.55, 3.43)	0.97 (0.79, 1.20)	1.21 (0.98, 1.49)	1.25 (1.01, 1.54)
**19F**	4.38 (3.73, 5.15)	4.51 (3.77, 5.38)	3.69 (3.17, 4.30)	0.97 (0.76, 1.24)	1.19 (0.95, 1.48)	1.22 (0.97, 1.54)
**22F**	4.26 (3.68, 4.93)	3.82 (3.22, 4.53)	3.55 (3.05, 4.14)	1.11 (0.89, 1.40)	1.20 (0.97, 1.48)	1.08 (0.86, 1.35)
**23F**	1.40 (1.15, 1.72)	1.35 (1.14, 1.58)	1.28 (1.06, 1.55)	1.04 (0.81, 1.35)	1.09 (0.83, 1.44)	1.05 (0.82, 1.35)
**33F**	1.34 (1.08, 1.66)	1.13 (0.94, 1.35)	0.92 (0.75, 1.13)	1.19 (0.90, 1.58)	1.46 (1.08, 1.96)	1.22 (0.93, 1.61)

GMC: Geometric mean concentration, N = Number of participants. * Serotype-specific IgG GMCs were expressed in µg/mL.

## Data Availability

All relevant data are included in the manuscript. Additional reasonable requests may be directed to the corresponding author.

## Supplementary Materials

The following supporting information can be downloaded at: https://www.mdpi.com/article/10.3390/vaccines14060464/s1, S1: Inclusion and exclusion criteria; S2: Study sites, ethics committees and SAATHI investigators; S3: Composition of study vaccines; S4: Serology assays and centres; S5: Summary of AEs by SOC & PT from dose 1 to 28 days post 3 dose primary series (Day 84)—Safety Population (N = 2600); S6: Summary of unsolicited adverse events by SOC & PT from dose 1 to Day 84—Safety Population (N = 2600); S7: Summary of adverse events by SOC & PT post Day 84 to 6 months follow-up period—Safety Population (N = 2600); S8: Summary of serious adverse events (SAEs) by SOC & PT from dose Day 0 to Day 84 and during 6-month follow-up period—Safety Population (N = 2600); S9: Summary of serious adverse events (SAEs) by SOC & PT post Day 84 to 6-month follow-up period—Safety Population (N = 2600); S10: Forest plot for non-inferiority analysis by (A) Percentage differences in seroprotection/seroconversion rates at Day 84 (test minus control) with 95% CI (left), (B) Geometric mean ratios at Day 84 (test/control) with 95% CI (right); S11: Forest plot for equivalence analysis by serotype specific anti-pneumococcal capsular polysaccharide IgG GMC ratios at Day 84 (lot1/lot2, lot1/lot3, lot2/lot3) with 95% CI. S12: Table of Serotype-specific IgG GMC ratios for three lots of BE-PCV14 at Day 84, normalized to Lot 1 as the reference lot.
