# Supplementary material for: Safety, Immunogenicity of Co-Administered Vaccines, and Lot-to-Lot Consistency of a 14-Valent Pneumococcal Conjugate Vaccine (PNEUBEVAX 14®) Administered at 6–10–14 Weeks in Healthy Infants: A Multicenter, Phase IV Trial
Source: Vaccines (Basel). 2026 May 22;14(6):464. doi: 10.3390/vaccines14060464 (PMC13307665; doi:10.3390/vaccines14060464)
Supplement: Supplementary file 1 [file vaccines-14-00464-s001.zip › vaccines-4264133-supplementary.pdf]

Supplementary material

S1. INCLUSION AND EXCLUSION CRITERIA:

Inclusion Criteria:

Participants who had met all the following inclusion criteria were selected for study participation:

1. Healthy pneumococcal conjugate vaccine-naïve (PCV-naive) infants as established by medical history and clinical assessment before entering into the study. PCV-naïve infants are those who have not been previously vaccinated with any licensed or investigational pneumococcal vaccine (only for Safety arm).
2. Healthy pneumococcal conjugate vaccine-naïve (PCV-naive), Pentavalent vaccine (DTwP-rHepB-Hib) naïve, IPV vaccine naïve and live attenuated Rotavirus vaccine naïve infants as established by medical history and clinical assessment before entering into the study. PCV/Pentavalent/IPV/Rotavirus vaccine-naïve infants are those who have not been previously vaccinated with any licensed or investigational PCV/Pentavalent/IPV/Rotavirus vaccines (only for immunogenicity arm).
3. Infants between 6-8 weeks of age (42-56 days, both days inclusive) of either gender, at the time of first dose of vaccination.
4. Healthy Infants with body weight ≥ 3300 gms at the time of screening.
5. Participants' parent(s)/ LAR(s) who, in the opinion of the investigator, can and will comply, with the requirements of the protocol (e.g. completion of the diary cards, return for follow-up visits, with access to a consistent means of telephone contact, either residential landline or mobile).
6. Participant's parent(s)/LAR(s) willing to provide written or thumb printed informed consent (including audio visual recording of consent process) prior to performing any study specific procedure
7. Infants with a minimal vaccination status for their age at the time of enrolment ("minimal" defined as single dose of only BCG, Hepatitis B &/or Polio vaccine prior to enrolment).

Exclusion Criteria:

Participants who had met any of the following exclusion criteria were not enrolled in the study:

1. Child in care, defined as a child who has been placed under the control or protection of an agency, organisation, institution or entity by the courts, the government or a government body, acting in accordance with powers conferred on them by law or regulation. The definition of a child in care can include a child cared for by foster parents or living in a care home or institution, provided that the arrangement falls within the definition above. The definition of a child in care does not include a child who was adopted or has an appointed legal guardian.
2. Evidence of previous Streptococcus pneumoniae infection or pneumococcal vaccination.
3. Evidence of previous or intercurrent or known exposure to diphtheria, tetanus, pertussis, hepatitis B, poliomyelitis, H. influenzae type b and rotavirus diseases (immunogenicity arm only)
4. Use of any investigational or non-registered product (drug or vaccine) during the period starting 30 days before the administration of study vaccine (Day -29 to Day 0), or planned use during the study period other than the study vaccine.
5. Any medical condition that in the judgment of the investigator would make intramuscular injection unsafe (eg. coagulation abnormalities).
6. Concurrently participating in another clinical study, at any time during the study period, in which the participant has been or was exposed to an investigational or a non-investigational vaccine/product (pharmaceutical product or device).
7. History of allergic disease or history of a serious reaction to any prior vaccination or known hypersensitivity likely to be exacerbated by any component of the study vaccines.
8. History of any neurological disorders, meningitis or seizures.
9. Infant who has had a sibling die of sudden infant death syndrome (SIDS) or die suddenly and without apparent other cause or preceding illness in the first year of life.
10. Infant is a direct descendant (child or grand-child) of any person employed by the Sponsor, the Contract Research Organization (CRO) or the Study Site (including the PI and study site personnel).
11. Acute disease and/or fever at the time of vaccination.
    - Fever was defined as the endogenous elevation of at least one measured body temperature of $\geq 38°C$ ($\geq 100.4°F$).

12. Acute or chronic, clinically significant pulmonary, cardiovascular, hepatic or renal functional abnormality, as determined by physical examination and Principal investigator judgement.

S2. STUDY SITES, ETHICS COMMITTEES AND SAATHI INVESTIGATORS

Site ID	Investigator and Study Site	EC Name	Approval Date
1.	Dr. Nagarjuna Chadalavada M.B.B.S & MD (Paediatrics) Aditya Multispecialty Hospital, 13-4-64, 4th Lane, Gunturvari Thota, Kothapeta, Guntur 522001, Andhra Pradesh, India.	IEC Aditya Multispeciality Hospital 13-3-52, 3rd Line, Gunturivari Thota, Kothapet, Beside Peoples Trauma Guntur, Andhra Pradesh -522001, India (ECR/1347/Inst/AP/2020)	24-08-23
2.	Dr. Atul Jindal M.B.B.S, DCH & MD (Paediatrics) All India Institute Of Medical Sciences (AIIMS), Gate No, 1, Great Eastern Rd, opposite Gurudwara, AIIMS Campus, Tatibandh, Raipur 492099, Chhattisgarh, India.	Institute Ethics Committee, Room no. 2103, 2nd Floor South Wing, Medical College Complex, Gate No. 5. All India Institute of Medical Sciences, Tatibandh, GE Road, Raipur, 492099, Chhattisgarh, India. (ECR/714/Inst/CT/2015/RR-21)	28-08-23
3	Dr. Bhagirathi Dwibedi M.B.B.S & MD (Paediatrics) All India Institute of Medical Sciences, Sijua, Patrapada, Bhubaneswar 751019, Odisha, India.	Institutional Ethics Committee- All India Institute of Medical Sciences, BBSR, Sijua P/O Patrapada, Bhubaneswar Khordha Orissa – 751019, India (ECR/534/Inst/OD/2014/RR-20)	06-02-24
4.	Dr. Malesh. K M.B.B.S, DNB & MD (Paediatrics) Bangalore Medical College and Research Institute, Fort, Krishna Rajendra Rd, Bengaluru 560002, Karnataka, India.	Ethics Committee Of Bangalore Medical College And Research Institute Fort K R Road (Bangalore) Urban Karnataka – 560002, India (ECR/302/Inst/KA/2013/RR-20)	14-02-24

Site ID	Investigator and Study Site	EC Name	Approval Date
5.	Dr. Kiritkumar Jesabhai Sisodia M.B.B.S & MD (Paediatrics)<br>BAPS Pramukh Swami Hospital, Shri Pramukh Swami Maharaj Marg, Adajanchar rasta. Adajan. Surat- 395009, Gujarat, India.	BAPS Hospital Institutional Ethics Committee<br>BAPS Pramukh Swami Hospital, Adajan Cross Road, Surat Gujarat - 395009 India<br>(ECR/639/Inst/GJ/2014/RR-20)	09-10-23
6.	Dr. Chinmayi Joshi M.B.B.S, MD (Paediatrics)<br>Belagavi Institute Of Medical Sciences, Dr B R Ambedkar Rd, Sadashiv Nagar, Belgaum 590019, Karnataka, India.	Institutional Ethics Committee-<br>Belagavi Institute Of Medical Sciences, Dr B R Ambedkar Road, Belagavi (Belgaum) Karnataka - 590001 India<br>(ECR/801/Inst/KA/2016/RR-20)	31-08-23
7.	Dr. Ramesh. M M.B.B.S, DCH & MD (Paediatrics)<br>BGS Global Institute of Medical Sciences, Dr.Vishnuvardhan Rd, Kengeri, Bengaluru 560060, Karnataka, India.	Institutional Ethics Committee, Department of Community Medicine, Room No:01, Second floor, College Building, BGS Global Institute of Medical Sciences 67 BHS Health and Education City Uttarahalli Road Kengeri, Bangalore South Karnataka-560060, India<br>(ECR/1307/Inst/KA/2019)	31-08-23
8.	Dr. M. Alexander M.B.B.S & M.D (Paediatrics)<br>Chettinad Hospital And Research Institute, SH 49A, Kelambakkam, Chennai 603103, Tamil Nadu, India.	Institutional Human Ethics Committee Chettinad Academy Of Research And Education<br>Rajiv Gandhi Salai Kelambakkam, Kanchipuram Tamil Nadu – 603103, India<br>(ECR/212/Inst/TN/2013/RR-19)	26-03-24
9.	Dr. Kishori Sharan Agarwal M.B.B.S & DNB (Paediatrics)<br>Chirayu Hospital, Kalwar Rd, Hathoj, Jaipur 302012, Rajasthan, India.	IEC Chirayu Hospital A Unit of KSCH Pvt. Ltd. Chirayu Hospital, Kalwar Road Hathoj Jaipur Rajasthan - 302012 India<br>(ECR/1582/Inst/RJ/2021)	15-09-23

Site ID	Investigator and Study Site	EC Name	Approval Date
10.	Dr. Girish. P. Charde M.B.B.S & DCH<br>Gillurkar Multispeciality Hospital, 20, Reshimbag, Umred Road, Nagpur 440009, Maharashtra, India.	Gillurkar Hospital Ethics Committee, Plot no. 20, Reshimbag, Umred Road, Nagpur-440009, Maharashtra, India (ECR/1374/Inst/MH/2020)	18-09-23
11.	Dr, Manish Narang M.B.B.S & MD (Paediatrics)<br>GTB Hospital, Delhi, Tahirpur Rd, GTB Enclave, Dilshad Garden, Delhi 110095, India.	Guru Teg Bahadur Hospital Ethics Committee-Guru Teg Bahadur Hospital Dilshad Garden Delhi East<br>Delhi - 110095 India<br>(ECR/510/Inst/DL/2014/RR-20)	18-09-23
12.	Dr. Jai Prakash Narayan M.B.B.S & M.D (Paediatrics)<br>JLN Medical College, Kala Bagh, Ajmer 305001, Rajasthan, India.	Institutional Ethics Committee, Jawahar Lal Nehru Medical College, Kala Bagh, Ajmer, Rajasthan – 305001, India<br>(ECR/1156/Inst/RJ/2018/RR-22)	20-09-23
13.	Dr. M.D. Ravi M.B.B.S, DCH & M.D (Paediatrics)<br>JSS Hospital, Mahatma Gandhi Road, Fort Mohalla, Mysuru-570004, Karnataka, India.	Institutional Ethics Committee, JSS Medical College, JSS Hospital, Sri Shivarathreeshwara Nagara, Mysuru (Mysore) Karnataka – 570015 India<br>(ECR/387/Inst/KA/2013/RR-22)	12-02-24
14.	Dr. B. S. Chakravarthy M.B.B.S & M.D (Paediatrics)<br>King George Hospital Collectorate Junction, Maharanipeta, Visakhapatnam – 530002, Andhra Pradesh, India	IEC King George Hospital, Maharanipeta Collectorate Office Junction, Visakhapatnam -530002, Andhra Pradesh, India.<br>(ECR/197/Inst/KGH/2013/RR-20)	30-10-23
15.	Dr. N.S. Mahantshetti M.B.B.S & M.D (Paediatrics)<br>KLEs Prabhakar Kore Hospital & Medical Research Centre, Nehru Nagar, Belagavi - 590010, Karnataka, India.	Institutional Ethics Committee, KLE University Dr.PK Hospital and MRC Nehru Nagar Belagavi (Belgaum), Karnataka - 590010 India<br>(ECR/211/Inst/KA/2013/RR-19)	13-02-24

Site ID	Investigator and Study Site	EC Name	Approval Date
16.	Dr. Anju J.K. Mehrotra M.B.B.S & DCH (Paediatrics)<br>Latha Mangeshkar Hospital Maharajbagh Rd, Sitabuldi, Nagpur - 440012, Maharashtra, India.	Institutional Ethics Committee<br>NKP Salve Institute of Medical Sciences, Digdoh Hills Hingna road Nagpur Maharashtra - 440019 India<br>(ECR/88/Inst/MH/2013/RR-19)	29-02-24
17.	Dr. Jog Pramod Prabhakar M.B.B.S, M.D. & DNB (Paediatrics), Medipoint Hospital, 3rd Floor, Nagaras Road, DP Road, Near Kumar Padmalaya, Aundh, Pune 411007, Maharashtra, India.	Penta-Med Ethics Committee-Medipoint Hospitals Pvt. Ltd 241/1, New D.P.Road, Near Sai Heritage, Aundh Pune Maharashtra -411007 India<br>(ECR/357/Inst/MH/2013/RR-20)	31-08-23
18.	Dr. Prashanth. M.V M.B.B.S & MD (Paediatrics)<br>Medstar Speciality Hospital, 641/17/1/3, Kodigehalli Main Rd, Sahakar Nagar, Sanjeevini Nagar, Bengaluru 560092, Karnataka, India.	Medstar Speciality Hospital Ethics Committee<br>Medstar Speciality Hospital<br>N0 614, 17/1/3, Kodigehalli Main Road Sahakarnagar Bengaluru, Urban Karnataka - 560092 India<br>(ECR/1324/Inst/KA/2019)	02-09-23
19.	Dr. Ramanath Andy Karayar M.B.B.S, DCH & MD (Paediatrics) Panimalar Medical College Hospital & Research Institute, Varadharajapuram, Chennai Outer Ring Rd, Poonamallee, Chennai 600123 Tamil Nadu, India.	PMCHRI-IHEC<br>Panimalar Medical College Hospital & Research Institute, Varadharajapuram Poonamallee, Chennai, Tiruvallur, Tamil Nadu – 600123 India.<br>(ECR/1399/Inst/TN/2020)	22-08-23
20.	Dr. B. Sudhakar M.B.B.S & DCH, Priya Children's Hospital, D.No. 57, 12-3, Panta Kaluva Rd, Maruti Colony, New P & T Colony, Patamata, Vijayawada 520010, Andhra Pradesh, India.	Institutional Ethics Committee Anu Hospitals, Kovelamudivari Street, Suryaraopet Vijayawada, Krishna Andhra Pradesh – 520002, India<br>(ECR/1049/Inst/AP/2018/RR-21)	28-08-23
21.	Dr. Savita Verma M.B.B.S & M.D (Pharmacology)	Institutional Ethics Committee, PGIMS UHS Rohtak Pt. BD Sharma, Post	11-08-23

Site ID	Investigator and Study Site	EC Name	Approval Date
	PT. B D Sharma Post Graduate Institute of Medical Sciences & Hospital Rohtak 124001, Haryana, India	Graduate Institute of Sciences, Rohtak Haryana – 124001, India (ECR/293/Inst/HR/2013/RR-19)	
22.	Dr. Prema. R M.B.B.S, DCH & DNB (Paediatrics) Rajarajeshwari Hospital, No.202, Kambipura, Mysore Road, Bengaluru 560 074, Karnataka, India.	Institutional Ethics Committee, Rajarajeshwari Medical College and Hospital, No.202, Kambipura, Mysore Road, Bengaluru - 560074 Karnataka, India. (ECR/361/Inst/ML/2022)	26-10-23
23.	Dr. Pareshkumar. A. Thakkar M.B.B.S, MD & DNB (Paediatrics) SSG General Hospital, Anandpura, Vadodara 390001, Gujarat, India.	Institutional Ethics Committee for Human Research, Medical College, Baroda Anandpura Vadodara, Gujarat, 390001, India. (ECR/85/Inst/GJ/2013/RR-19)	13-12-23
24.	Dr. Madhukar Pandey M.B.B.S, MD (Paediatrics) Subham Sudbhawana superspeciality Hospital, B31/80,23-Bhogabir, Lanka Varanasi- 221005, Uttar Pradesh, India.	Shubham Sudbhawana Super. Hosp. Ethics Committee, Shubham Sudbhawana Superspeciality Hospital B 31/80, 23B - Bhogabeer, Lanka, Varanasi, 221005, U.P. India. (ECR/667/Inst/UP/2014/RR-20)	12-08-23
25.	ESIC Medical College & Hospital, Room No. 4007, 4th Floor, NH-3 behind BK Hospital New Industrial Town, Faridabad-121001, Haryana, India.	Institutional Ethics Committee for ESIC Faridabad ESIC Medical College and Hospital NH-3, NIT, Behind BK Hospital, Faridabad 121001, Haryana, India. (ECR/1539/Inst/HR/2021)	05-03-24
26.	All India Institute of Medical Sciences Gorakhpur Department of Pharmacology and Therapeutics 3rd, AIMS Kunraghat Gorakhpur, Uttar Pradesh, 273008, India.	Institutional Human Ethics Committee All India Institute Of Medical Sciences, Gorakhpur Opp Army Public School Kunraghat Gorakhpur Gorakhpur Uttar Pradesh -	16-02-24

Site ID	Investigator and Study Site	EC Name	Approval Date
		273008India (ECR/1476/Inst/UP/2020)	
27.	D. Y. Patil Hospital and Research Centre, Ground floor of Department of Paediatrics, Ayyappa Temple Rd, Dr D Y Patil Vidyanagar, Sector 5, Nerul, Navi Mumbai, Maharashtra 400706, India.	Institutional Ethics Committee D Y Patil Medical College Sector 5 Nerul Navi Mumbai Thane Maharashtra 400706 India.<br>(ECR/195/Inst/MH/2013/RR-19)	18-03-24
28.	KEM Hospital Research Centre Vadu Rural Health Program At Post Vadu (Budruk), Taluka Shirur District, Pune-412216, Maharashtra India.	KEM Hospital Research Centre Ethics Committee<br>KEM Hospital Research Centre<br>TDH Building, 3rd floor, Room No - 303, Sardar Moodliar Road, Rasta Peth Pune, Maharashtra - 411011 India.<br>(ECR/272/Inst/MH/2013/RR-22)	19-04-24
29.	Maharaja Agrasen Superspeciality Hospital Clinical Research Department, Sec 7, Central Spin, Vidyadhar Nagar, Jaipur-302039, India.	IEC, Maharaja Agrasen Hospital, Maharaja Agrasen Superspeciality Hospital<br>Central Spine, Agrasen Aspatal Marg Sector 7, Vidhyadhar Nagar, Jaipur, Rajasthan – 302039, India.<br>(ECR/1222/Inst/RJ/2019/RR-22)	20-03-24
30.	New Leelamani Hospital, Civil Lines, 14/116, C-1, Parade Chauraha, Civil Lines, Kanpur, Uttar Pradesh – 208001, India.	Institutional Ethics Committee-Leelamani Hospital New Leelamani Hospital14/116 B, C, C-1D, 1 Civil Line Kanpur, Nagar Uttar Pradesh - 208001 India.<br>(ECR/1696/Inst/UP/2022)	20-03-24
31.	St. Theresa’s Hospital (STH), 1st Floor, Room No. 05, Erragadda Main Road, Czech Colony Sanath Nagar, Hyderabad - 500038, Telangana, India.	Ethics committee St Theresa’s Hospital Sanathnagar, Opp. Erragadda Raitu Bazar Hyderabad, Telangana - 500018 India. (ECR/230/Inst/AP/2013/RR-22)	27-03-24

S3. COMPOSITION OF STUDY VACCINES

BE-PCV14 (PNEUBEVAX 14®): Lot1: 421500223A, Lot2: 421500323A, Lot3: 421500423A

Each dose of (0.5mL) contains:

Pneumococcal polysaccharide serotype 1………………3.0 µg

Pneumococcal polysaccharide serotypes 3, 4, 5, 7F, 9V,

14, 18C, 19A, 19F, 22F, 23F and 33F (each)……………2.2 µg

Pneumococcal polysaccharide serotype 6B………………4.4 µg

Adsorbed on to Aluminium Phosphate, as Al+++………≤0.75 mg

2-Phenoxy ethanol ………………………………………4 mg

Polysaccharide conjugated to 20-50 µg of CRM_{197}

PCV13 (Prevenar13®) : Pneumococcal Polysaccharide Conjugate Vaccine (Adsorbed) I.P., 13-valent

Each single dose (0.5ml) pre-filled syringe contains

2.2 µg of saccharide for serotype 1, 3, 4, 5, 6A, 7F, 9V, 14, 18C, 19A, 19F and 23F and 4.4 µg of saccharide for serotype 6B, conjugated to about 32µg of CRM_{197} protein and adsorbed on aluminium phosphate (0.125 mg aluminium)

Inactivate ingredients: Sodium chloride, Succinic Acid, Polysorbate 80 and Water for injection.

DTwP-HepB-IPV-Hib (HEXASIIL®): Diphtheria, Tetanus, Pertussis (Whole Cell), Hepatitis-B (rDNA), Poliomyelitis (Inactivated) and *Haemophilus influenzae* type b Conjugate Vaccine (Adsorbed) I.P.

Each dose of 0.5mL contains

Diphtheria Toxoid……………………………………...…. ≥ 30

Tetanus Toxoid ………………………………….………… ≥ 40 IU

B. pertussis (whole cell) ……………………………….... ≥ 4 IU

HBsAg (rDNA) ………………………………………………...15 mcg

Inactivated Polio Vaccine (Salk strains grown on vero cells)

Type 1 (Mahoney strain) …………………………………………. 40 DU

Type 2 (MEF-1 strain) ……………………………………………. 8 DU

Type 3 (Saukett strain) ……………………………….……………32 DU

Hib (PRP) …………………………………………………………...10 mcg
conjugate to TT (carrier protein) ……………………………………19 to 33 mcg
Aluminium content Al+++ (as Aluminium Phosphate gel) ……..…. ≤1.25 mg
2-Phenoxyethanol ………………………………………………..…. 0.5%
0.9% Sodium Chloride (Normal saline) …………………………... q.s.

Rotavirus (ROTAVAC 5D®): Rotavirus Vaccine (Live Attenuated, Oral) IP

Each dose of 0.5 mL contains:
Vero cell derived Rotavirus 116E bulk, Live attenuated ………… NLT $10^{5.0}$ FFU
Neomycin Sulphate IP…………………………………………… 15 µg
Kanamycin Acid Sulphate IP …………………………………... 15 µg
Sucrose IP……………………………………………………… 0.25 gms
Trehalose BP…………………………………………………... 2.5 mg
Lactalbumin Hydrolysate (LAH) ………………………………… 2.5 mg
Human Albumin IP ……………………………………………….0.35%
Potassium Dihydrogen Orthophosphate BP ……………………... 1.65 mg
Dipotassium Hydrogen Orthophosphate BP …………………….. 10 mg
Trisodium Citrate Dihydrate IP …………………………………. 7.75 mg
Water for Injection IP …………………………………………… q.s.

S4. SEROLOGY ASSAYS AND CENTRES

Serotype-specific anti-pneumococcal capsular polysaccharide IgG concentrations were measured using an enzyme-linked immunosorbent assay developed at Biological E Limited laboratories based on the WHO training manual for pneumococcal ELISA, with minor modifications. No commercial ELISA kit was used; therefore, kit manufacturer and catalogue number details are not applicable. The assay was validated for accuracy, precision, range, linearity, stability, and robustness. The 1st International Standard for Human Anti-pneumococcal Capsular Reference Serum, 007sp, supplied by the National Institute for Biological Standards and Control, UK, was used as the reference standard. This standard has assigned IgG concentrations for pneumococcal capsular serotypes. The validated quantitation range varied by serotype; the nominal upper and lower limits of quantitation were 100 ng/mL and 0.15625

ng/mL, respectively. Dilution-corrected antibody concentrations were calculated using a 4-parameter logistic regression model.

“Briefly, pre-immune and post-immune human serum samples, reference serum, and quality-control serum were pre-absorbed with CWPS Multi™ to remove cell-wall polysaccharide-reactive antibodies. The pre-absorbed sera were then added to ELISA plates coated with pneumococcal capsular polysaccharide of the respective serotype. Serotype-specific anti-pneumococcal IgG antibodies bound to the coated antigen were detected using goat anti-human IgG alkaline phosphatase conjugate. Following addition of pNPP chromogenic substrate, optical density was measured at 405 nm with 690 nm as the reference wavelength. The corrected optical density was calculated as OD405 − OD690 and was proportional to the amount of serotype-specific anti-pneumococcal IgG in the serum sample.

Immune responses to co-administered vaccines were assessed at designated laboratories using validated assays. IgG concentrations against diphtheria, tetanus, hepatitis B, *Haemophilus influenzae* type b, and pertussis antigens were measured at Dr. Dangs Laboratory, Delhi, India. Quantitative ELISA kits were used for anti-diphtheria, anti-tetanus, and anti-pertussis toxin IgG antibodies; anti-HBs antibody titers were measured using the Alinity i Anti-HBs Reagent Kit; anti-PRP antibodies were measured using the VaccZyme Human Anti-*Haemophilus influenzae* type b Enzyme Immunoassay Kit; neutralizing antibodies against poliovirus types 1, 2, and 3 were measured by microneutralization assay at the Clinical Serology Laboratory, Biological E. Limited; and anti-rotavirus IgA concentrations were estimated at the Central Research Laboratory, Kempegowda Institute of Medical Sciences, Bengaluru, India, using a VP6 IgA ELISA kit.

S5. SUMMARY OF AES BY SOC & PT FROM DOSE 1 TO 28 DAYS POST 3 DOSE PRIMARY SERIES (DAY 84) - SAFETY POPULATION (N=2600)

System Organ Class<br>Preferred Term<br>n (%) [95% CI] E	PNEUBEVAX 14<br>(N = 2300)	PREVENAR 13<br>(N = 300)
	606 (26.35)	74 (24.67)

System Organ Class<br>Preferred Term<br>n (%) [95% CI] E	PNEUBEVAX 14<br>(N = 2300)	PREVENAR 13<br>(N = 300)
No. of participants with at least one adverse event reported during Day 0 to Day 84	[24.59, 28.19] 1253	[20.13, 29.84] 93
Ear and labyrinth disorders	3 (0.13)<br>[0.04, 0.38] 3	0<br>NE 0
Ear pain	2 (0.09)<br>[0.02, 0.32] 2	0<br>NE 0
Otorrhoea	1 (0.04)<br>[0.01, 0.25] 1	0<br>NE 0
Eye disorders	1 (0.04)<br>[0.01, 0.25] 1	0<br>NE 0
Lacrimation increased	1 (0.04)<br>[0.01, 0.25] 1	0<br>NE 0
Gastrointestinal disorders	28 (1.22)<br>[0.84, 1.75] 31	0<br>NE 0
Abdominal pain	1 (0.04)<br>[0.01, 0.25] 1	0<br>NE 0
Diarrhoea	22 (0.96)<br>[0.63, 1.44] 23	0<br>NE 0
Vomiting	7 (0.30)<br>[0.15, 0.63] 7	0<br>NE 0
General disorders and administration site conditions	597 (25.96)<br>[24.21, 27.79] 1152	71 (23.67)<br>[19.21, 28.79] 90
Crying	1 (0.04)<br>[0.01, 0.25] 1	0<br>NE 0
Injection site erythema	105 (4.57)<br>[3.79, 5.50] 111	15 (5.00)<br>[3.05, 8.08] 16
Injection site induration	41 (1.78)<br>[1.32, 2.41] 46	0<br>NE 0
Injection site pain	253 (11.00)<br>[9.79, 12.34] 313	26 (8.67)<br>[5.98, 12.40] 26
Injection site swelling	147 (6.39)<br>[5.46, 7.47] 175	12 (4.00)<br>[2.30, 6.86] 15
Irritability postvaccinal	68 (2.96)<br>[2.34, 3.73] 100	1 (0.33)<br>[0.06, 1.86] 1
Pyrexia	324 (14.09)<br>[12.73, 15.57] 406	31 (10.33)<br>[7.38, 14.29] 32
Infections and infestations	24 (1.04)<br>[0.70, 1.55] 29	2 (0.67)<br>[0.18, 2.40] 2
Folliculitis	1 (0.04)<br>[0.01, 0.25] 1	0<br>NE 0
Gastroenteritis	2 (0.09)<br>[0.02, 0.32] 2	0<br>NE 0
Impetigo	1 (0.04)<br>[0.01, 0.25] 1	0<br>NE 0
Nasopharyngitis	3 (0.13)<br>[0.04, 0.38] 3	2 (0.67)<br>[0.18, 2.40] 2
Pneumonia viral	1 (0.04)	0

System Organ Class<br>Preferred Term<br>n (%) [95% CI] E	PNEUBEVAX 14<br>(N = 2300)	PREVENAR 13<br>(N = 300)
	[0.01, 0.25] 1	NE 0
Rhinitis	12 (0.52)<br>[0.30, 0.91] 13	0<br>NE 0
Tinea faciei	1 (0.04)<br>[0.01, 0.25] 1	0<br>NE 0
Upper respiratory tract infection	7 (0.30)<br>[0.15, 0.63] 7	0<br>NE 0
Metabolism and nutrition disorders	24 (1.04)<br>[0.70, 1.55] 27	0<br>NE 0
Decreased appetite	22 (0.96)<br>[0.63, 1.44] 25	0<br>NE 0
Dehydration	2 (0.09)<br>[0.02, 0.32] 2	0<br>NE 0
Nervous system disorders	3 (0.13)<br>[0.04, 0.38] 3	0<br>NE 0
Somnolence	3 (0.13)<br>[0.04, 0.38] 3	0<br>NE 0
Respiratory, thoracic and mediastinal disorders	2 (0.09)<br>[0.02, 0.32] 2	1 (0.33)<br>[0.06, 1.86] 1
Cough	2 (0.09)<br>[0.02, 0.32] 2	1 (0.33)<br>[0.06, 1.86] 1
Skin and subcutaneous tissue disorders	4 (0.17)<br>[0.07, 0.45] 5	0<br>NE 0
Dermatitis contact	1 (0.04)<br>[0.01, 0.25] 1	0<br>NE 0
Urticaria	3 (0.13)<br>[0.04, 0.38] 4	0<br>NE 0

S6. SUMMARY OF UNSOLICITED ADVERSE EVENTS BY SOC & PT FROM DOSE 1 TO DAY 84 - SAFETY POPULATION (N=2600)

System Organ Class<br>Preferred Term<br>n (%) [95% CI] E	PNEUBEVAX 14<br>(N = 2300)	PREVENAR 13<br>(N = 300)
No. of participants with at least one unsolicited AE during Day 0 to Day 84	40 (1.74)<br>[1.28, 2.36] 63	3 (1.00)<br>[0.34, 2.90] 3
Ear and labyrinth disorders	3 (0.13)<br>[0.04, 0.38] 3	0 (0)<br>[NE] 0
Ear pain	2 (0.09)<br>[0.02, 0.32] 2	0 (0)<br>[NE] 0
Otorrhoea	1 (0.04)<br>[0.01, 0.25] 1	0 (0)<br>[NE] 0
Eye disorders	1 (0.04)<br>[0.01, 0.25] 1	0 (0)<br>[NE] 0
Lacrimation increased	1 (0.04)<br>[0.01, 0.25] 1	0 (0)<br>[NE] 0
Gastrointestinal disorders	9 (0.39)	0 (0)

System Organ Class<br>Preferred Term<br>n (%) [95% CI] E	PNEUBEVAX 14<br>(N = 2300)	PREVENAR 13<br>(N = 300)
	[0.21, 0.74] 11	[NE] 0
Abdominal pain	1 (0.04)<br>[0.01, 0.25] 1	0 (0)<br>[NE] 0
Diarrhoea	8 (0.35)<br>[0.18, 0.68] 9	0 (0)<br>[NE] 0
Vomiting	1 (0.04)<br>[0.01, 0.25] 1	0 (0)<br>[NE] 0
General disorders and administration site conditions	11 (0.48)<br>[0.27, 0.85] 11	0 (0)<br>[NE] 0
Injection site erythema	1 (0.04)<br>[0.01, 0.25] 1	0 (0)<br>[NE] 0
Injection site swelling	1 (0.04)<br>[0.01, 0.25] 1	0 (0)<br>[NE] 0
Pyrexia	9 (0.39)<br>[0.21, 0.74] 9	0 (0)<br>[NE] 0
Infections and infestations	24 (1.04)<br>[0.70, 1.55] 29	2 (0.67)<br>[0.18, 2.40] 2
Folliculitis	1 (0.04)<br>[0.01, 0.25] 1	0 (0)<br>[NE] 0
Gastroenteritis	2 (0.09)<br>[0.02, 0.32] 2	0 (0)<br>[NE] 0
Impetigo	1 (0.04)<br>[0.01, 0.25] 1	0 (0)<br>[NE] 0
Nasopharyngitis	3 (0.13)<br>[0.04, 0.38] 3	2 (0.67)<br>[0.18, 2.40] 2
Pneumonia viral	1 (0.04)<br>[0.01, 0.25] 1	0 (0)<br>[NE] 0
Rhinitis	12 (0.52)<br>[0.30, 0.91] 13	0 (0)<br>[NE] 0
Tinea faciei	1 (0.04)<br>[0.01, 0.25] 1	0 (0)<br>[NE] 0
Upper respiratory tract infection	7 (0.30)<br>[0.15, 0.63] 7	0 (0)<br>[NE] 0
Metabolism and nutrition disorders	2 (0.09)<br>[0.02, 0.32] 2	0 (0)<br>[NE] 0
Dehydration	2 (0.09)<br>[0.02, 0.32] 2	0 (0)<br>[NE] 0
Nervous system disorders	3 (0.13)<br>[0.04, 0.38] 3	0 (0)<br>[NE] 0
Somnolence	3 (0.13)<br>[0.04, 0.38] 3	0 (0)<br>[NE] 0
Respiratory, thoracic and mediastinal disorders	2 (0.09)<br>[0.02, 0.32] 2	1 (0.33)<br>[0.06, 1.86] 1
Cough	2 (0.09)<br>[0.02, 0.32] 2	1 (0.33)<br>[0.06, 1.86] 1
Skin and subcutaneous tissue disorders	1 (0.04)<br>[0.01, 0.25] 1	0 (0)<br>[NE] 0

System Organ Class<br>Preferred Term<br>n (%) [95% CI] E	PNEUBEVAX 14<br>(N = 2300)	PREVENAR 13<br>(N = 300)
Dermatitis contact	1 (0.04)<br>[0.01, 0.25] 1	0 (0)<br>[NE] 0

S7. SUMMARY OF ADVERSE EVENTS BY SOC & PT POST DAY 84 TO 6 MONTHS FOLLOW - SAFETY POPULATION (N=2600)

System Organ Class<br>Preferred Term<br>n (%) [95% CI] E	PNEUBEVAX 14<br>(N = 2300)	PREVENAR 13<br>(N = 300)
No. of Participants with at least one AE during Day 84 to the booster dose visit	59 (2.57)<br>[1.99, 3.29] 74	5 (1.67)<br>[0.71, 3.84] 5
Blood and lymphatic system disorders	1 (0.04)<br>[0.01, 0.25] 1	0 (0)<br>[NE] 0
Anaemia	1 (0.04)<br>[0.01, 0.25] 1	0 (0)<br>[NE] 0
Ear and labyrinth disorders	1 (0.04)<br>[0.01, 0.25] 1	0 (0)<br>[NE] 0
Ear pain	1 (0.04)<br>[0.01, 0.25] 1	0 (0)<br>[NE] 0
Gastrointestinal disorders	18 (0.78)<br>[0.50, 1.23] 18	1 (0.33)<br>[0.06, 1.86] 1
Abdominal pain	1 (0.04)<br>[0.01, 0.25] 1	1 (0.33)<br>[0.06, 1.86] 1
Constipation	1 (0.04)<br>[0.01, 0.25] 1	0 (0)<br>[NE] 0
Diarrhoea	6 (0.26)<br>[0.12, 0.57] 6	0 (0)<br>[NE] 0
Vomiting	10 (0.43)<br>[0.24, 0.80] 10	0 (0)<br>[NE] 0
General disorders and administration site conditions	21 (0.91)<br>[0.60, 1.39] 21	3 (1.00)<br>[0.34, 2.90] 3
Pyrexia	21 (0.91)<br>[0.60, 1.39] 21	3 (1.00)<br>[0.34, 2.90] 3
Infections and infestations	19 (0.83)<br>[0.53, 1.29] 24	0 (0)<br>[NE] 0
Conjunctivitis	1 (0.04)<br>[0.01, 0.25] 1	0 (0)<br>[NE] 0
Gastroenteritis	2 (0.09)<br>[0.02, 0.32] 2	0 (0)<br>[NE] 0
Nasopharyngitis	7 (0.30)<br>[0.15, 0.63] 7	0 (0)<br>[NE] 0
Rhinitis	1 (0.04)<br>[0.01, 0.25] 1	0 (0)<br>[NE] 0
Upper respiratory tract infection	11 (0.48)<br>[0.27, 0.85] 13	0 (0)<br>[NE] 0
Injury, poisoning and procedural complications	1 (0.04)<br>[0.01, 0.25] 1	0 (0)<br>[NE] 0

System Organ Class<br>Preferred Term<br>n (%) [95% CI] E	PNEUBEVAX 14<br>(N = 2300)	PREVENAR 13<br>(N = 300)
Burns second degree	1 (0.04)<br>[0.01, 0.25] 1	0 (0)<br>[NE] 0
Respiratory, thoracic and mediastinal disorders	5 (0.22)<br>[0.09, 0.51] 5	1 (0.33)<br>[0.06, 1.86] 1
Bronchospasm	1 (0.04)<br>[0.01, 0.25] 1	0 (0)<br>[NE] 0
Cough	3 (0.13)<br>[0.04, 0.38] 3	1 (0.33)<br>[0.06, 1.86] 1
Pneumonitis	1 (0.04)<br>[0.01, 0.25] 1	0 (0)<br>[NE] 0
Skin and subcutaneous tissue disorders	3 (0.13)<br>[0.04, 0.38] 3	0 (0)<br>[NE] 0
Rash	2 (0.09)<br>[0.02, 0.32] 2	0 (0)<br>[NE] 0
Urticaria	1 (0.04)<br>[0.01, 0.25] 1	0 (0)<br>[NE] 0

S8. SUMMARY OF SERIOUS ADVERSE EVENTS (SAES) BY SOC & PT FROM DOSE DAY 0 TO DAY 84 AND DURING 6-MONTH FOLLOW-UP PERIOD - SAFETY POPULATION (N=2600)

System Organ Class<br>Preferred Term<br>n (%) [95% CI] E	PNEUBEVAX 14<br>(N = 2300)	Prevenar 13<br>(N = 300)
No. of participants with at least one serious adverse event	4 (0.22)<br>[0.09, 0.51] 7	0 (0%)<br>[NE] 0
Infections and infestations	4 (0.17)<br>[0.07, 0.45] 4	0 (0%)<br>[NE] 0
Pneumonia viral	1 (0.04)<br>[0.01, 0.25] 1	0 (0%)<br>[NE] 0
Metabolism and nutrition disorders	2 (0.09)<br>[0.02, 0.32] 2	0 (0%)<br>[NE] 0
Dehydration	2 (0.09)<br>[0.02, 0.32] 2	0 (0%)<br>[NE] 0

S9. SUMMARY OF SERIOUS ADVERSE EVENTS (SAES) BY SOC & PT POST DAY 84 TO 6-MONTHS FOLLOW-UP - SAFETY POPULATION (N=2600)

System Organ Class<br>Preferred Term<br>n (%) [95% CI] E	PNEUBEVAX 14<br>(N = 2300)	Prevenar 13<br>(N = 300)
No. of participants with at least one serious adverse event	1 (0.04%)<br>[0.01, 0.25] 1	0 (0%)<br>[NE] 0

System Organ Class<br>Preferred Term<br>n (%) [95% CI] E	PNEUBEVAX 14<br>(N = 2300)	Prevenar 13<br>(N = 300)
Respiratory, thoracic and mediastinal disorders	1 (0.04%)<br>[0.01, 0.25] 1	0 (0%)<br>[NE] 0
Pneumonitis	1 (0.04%)<br>[0.01, 0.25] 1	0 (0%)<br>[NE] 0

S10. FOREST PLOT FOR NON-INFERIORITY ANALYSIS BY A. PERCENTAGE DIFFERENCES IN SEROPROTECTION/SEROCONVERSION RATES AT DAY 84 (TEST MINUS CONTROL) WITH 95% CI (LEFT), B. GEOMETRIC MEAN RATIOS AT DAY 84 (TEST/CONTROL) WITH 95% CI (RIGHT)

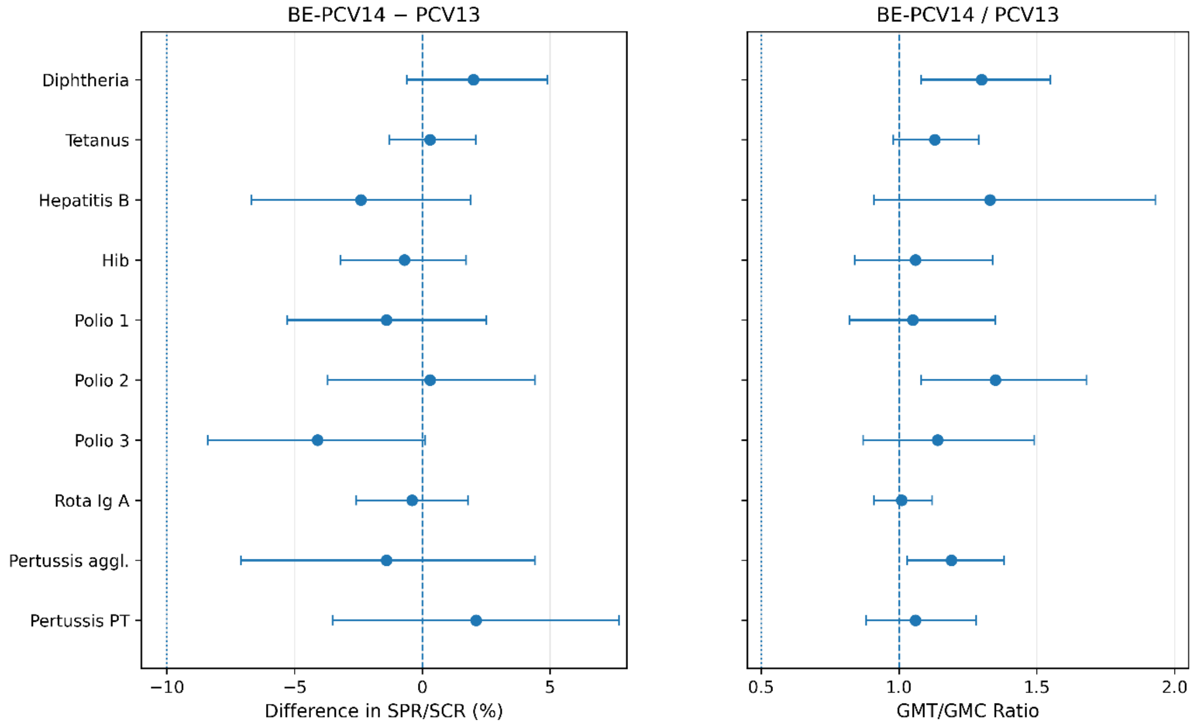

−10: non-inferiority margin

0.5: non-inferiority margin

S11. FOREST PLOT FOR EQUIVALENCE ANALYSIS BY SEROTYPE SPECIFIC ANTI-PNEUMOCOCCAL CAPSULAR POLYSACCHRIDE IgG GMC RATIOS AT DAY 84 (LOT1/LOT2, LOT1/LOT3, LOT2/LOT3) WITH 95% CI

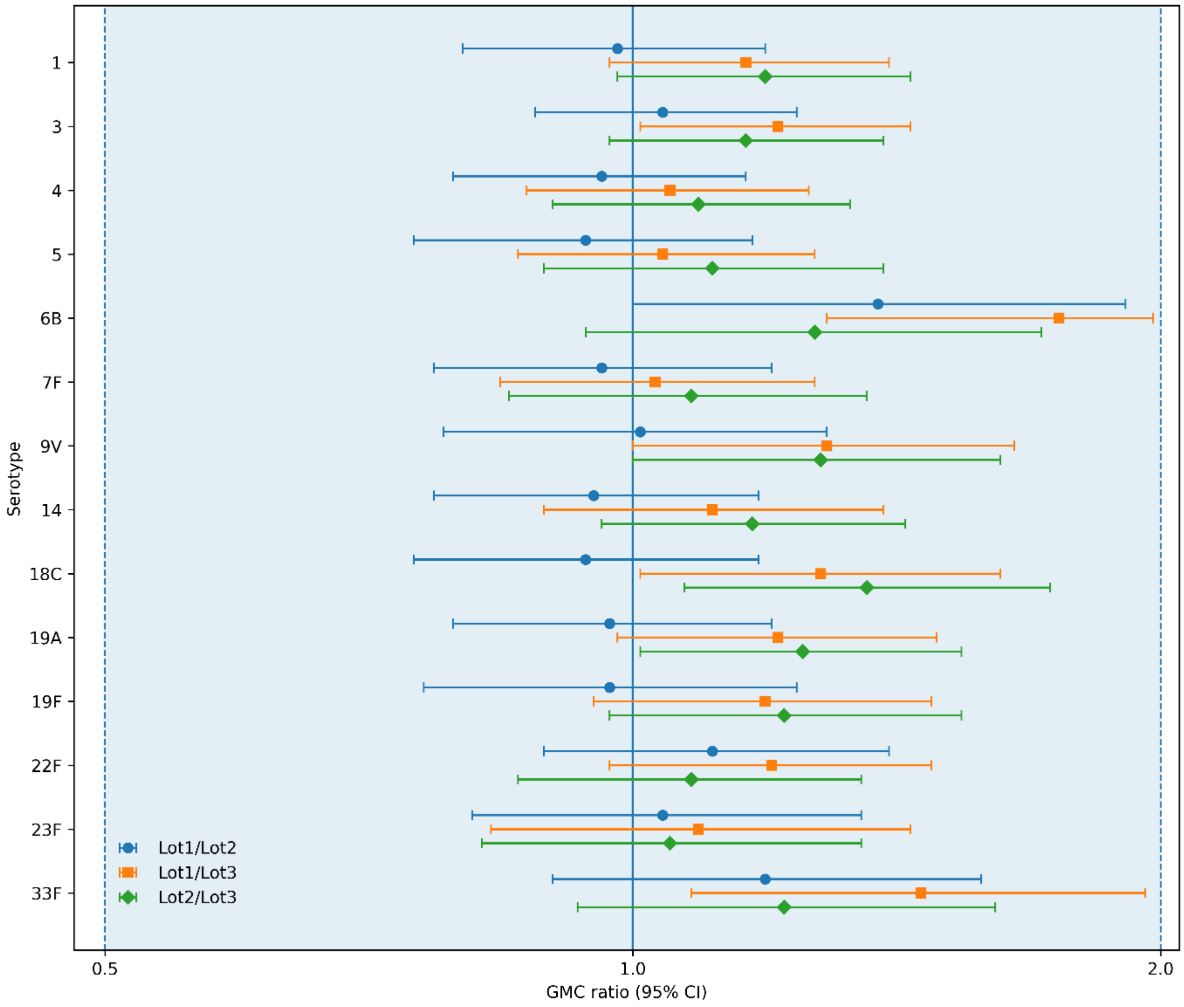

Dashed vertical lines indicate equivalence margins (0.5 to 2.0); solid vertical line indicates ratio = 1.0.

S12: Table of Serotype-specific IgG GMC ratios for three lots of BE-PCV14 at Day 84, normalized to Lot 1 as the reference lot

Serotype	Lot 1 / Lot1	Lot 2 / Lot 1 (95% CI)	Lot 3 / Lot 1 (95% CI)
1	1.00	1.02 (0.84, 1.25)	0.86 (0.71, 1.03)
3	1.00	0.96 (0.81, 1.14)	0.83 (0.69, 0.99)
4	1.00	1.04 (0.86, 1.27)	0.95 (0.79, 1.15)
5	1.00	1.06 (0.85, 1.33)	0.96 (0.79, 1.16)
6B	1.00	0.72 (0.52, 1.00)	0.57 (0.51, 0.78)
7F	1.00	1.04 (0.83, 1.30)	0.97 (0.79, 1.19)
9V	1.00	0.99 (0.78, 1.28)	0.78 (0.61, 1.00)
14	1.00	1.05 (0.85, 1.30)	0.90 (0.72, 1.12)

Serotype	Lot 1 / Lot1	Lot 2 / Lot 1 (95% CI)	Lot 3 / Lot 1 (95% CI)
18C	1.00	1.06 (0.85, 1.33)	0.78 (0.62, 0.99)
19A	1.00	1.03 (0.83, 1.27)	0.83 (0.67, 1.02)
19F	1.00	1.03 (0.81, 1.32)	0.84 (0.68, 1.05)
22F	1.00	0.90 (0.71, 1.12)	0.83 (0.68, 1.03)
23F	1.00	0.96 (0.74, 1.23)	0.92 (0.69, 1.20)
33F	1.00	0.84 (0.63, 1.11)	0.68 (0.51, 0.93)

Note: Lot 1 was used as the reference lot for normalized presentation. This analysis was added to improve visualization of between-lot variability. The formal lot-to-lot equivalence assessment was based on prespecified pairwise comparisons among all three lots, with equivalence concluded if the two-sided 95% CIs for all GMC ratios were within 0.5 to 2.0.
